# Strong Alterations in the Sphingolipid Profile of Chickens Fed a Dose of Fumonisins Considered Safe

**DOI:** 10.3390/toxins13110770

**Published:** 2021-10-30

**Authors:** Didier Tardieu, Maria Matard-Mann, Pi Nyvall Collén, Philippe Guerre

**Affiliations:** 1 National Veterinary School of Toulouse, ENVT, Université de Toulouse, F-31076 Toulouse, France; didier.tardieu@envt.fr; 2 Olmix S.A., ZA du Haut du Bois, F-56580 Bréhan, France; mmatard@olmix.com (M.M.-M.); pnyvallcollen@olmix.com (P.N.C.)

**Keywords:** fumonisin, sphinganine, sphingosine, ceramide, sphingomyelin, sphingolipid

## Abstract

Fumonisins (FB) are mycotoxins known to exert most of their toxicity by blocking ceramide synthase, resulting in disruption of sphingolipid metabolism. Although the effects of FB on sphinganine (Sa) and sphingosine (So) are well documented in poultry, little information is available on their other effects on sphingolipids. The objective of this study was to analyze the effects of FB on the hepatic and plasma sphingolipidome in chickens. The first concern of this analysis was to clarify the effects of FB on hepatic sphingolipid levels, whose variations can lead to numerous toxic manifestations. The second was to specify the possible use of an alteration of the sphingolipidome as a biomarker of exposure to FB, in addition to the measurement of the Sa:So ratio already widely used. For this purpose, we developed an UHPLC MS/MS method that enabled the determination of 82 SL, including 10 internal standards, in chicken liver and plasma. The validated method was used to measure the effects of FB administered to chickens at a dose close to 20 mg FB1 + FB2/kg feed for 9 days. Significant alterations of sphingoid bases, ceramides, dihydroceramides, glycosylceramides, sphingomyelins and dihydrosphingomyelins were observed in the liver. In addition, significant increases in plasma sphinganine 1-phosphate, sphingosine 1-phosphate and sphingomyelins were observed in plasma. Interestingly, partial least-squares discriminant analysis of 11 SL in plasma made it possible to discriminate exposed chickens from control chickens, whereas analysis of Sa and So alone revealed no difference. In conclusion, our results show that the effects of FB in chickens are complex, and that SL profiling enables the detection of exposure to FB when Sa and So fail.

## 1. Introduction

Fumonisins are mycotoxins produced by fungi of the genus *Fusarium* that are found worldwide [1,2,3,4,5]. Among the different groups of fumonisins identified, fumonisin B (FB), and in particular fumonisin B1 (FB1) and fumonisin B2 (FB2), are the most widespread and the most frequently studied [6]. These compounds may be present in human food and in animal feed, where they cause a number of health problems. The toxic effects of FB vary with the animal species but also with the dose and duration of exposure [1,7,8]. In equids, leucoencephalomalacia, characterized by softening of the white matter of the brain, is observed, while pulmonary edema is observed in pigs. Hepatic lesions are described in the two species as well as in all other animal species, including poultry. FB1 is carcinogenic in rodents, where it causes renal tubular tumors in male rats and liver tumors in female mice [9]. In humans, exposure studies have shown that FB consumption is associated with increased incidence of esophageal cancer [10]. These studies led to the classification of FB in group 2B as “probably carcinogenic to humans” [11]. A tolerable daily intake of FB has been established for humans, while maximum tolerable levels of FB have been established in animal feed and in raw materials used for their manufacture [1,12].

The toxicity of FBs is mainly associated with their effects on sphingolipid synthesis. The structure of FB1 is similar to that of sphingoid bases (SB) and inhibits ceramide synthase, leading to strong inhibition of de novo synthesis of sphingolipids [8,13,14]. Interestingly, sphinganine (Sa) contents in cells, and sometimes sphingosine (So) contents, increase before the onset of clinical signs of mycotoxicosis [1,7,8]. Because the effects on Sa occur earlier and are stronger than those on So, the Sa:So ratio was formerly proposed as a biomarker of FB exposure in mammals, and is also an effective biomarker in avian species [8,15,16]. Because So is not obtained from the de novo synthesis of SB, the increase in So observed during exposure to FB is evidence that the toxin also affects the recycling of sphingolipids. Finally, not only SB and ceramide contents, but also glycosylceramides and sphingomyelins, and nearly all the metabolites derived from SB could be altered during FB exposure [8]. Even disruptions of sphingolipid metabolism are commonly reported in animals suffering from FB toxicity; the target organs and signs of toxicity vary considerably with the animal species, the dose, and the duration of exposure, and the reasons for these variations remain largely unknown [6,8]. In particular, alterations to sphingolipid metabolism are poorly documented in avian species even though these species are recognized as one on the most resistant to FB [1,8,17,18]. Fine characterization of the effects of FB on the sphingolipid profile is thus necessary to understand the different manifestations of the disease and the consequence of exposure to FB at an apparently safe dose in feed.

Different approaches based on LC-MS/MS with a triple quadrupole mass spectrometer in multiple reaction monitoring (MRM) mode have been developed for the quantitative analysis of sphingolipids [19,20,21,22]. Due to the complexity of sphingolipid metabolism, profiling of individual sphingolipids is necessary. However, as concentrations of sphingolipids can vary by one thousand and more, the quantitation of low- abundant species is sometimes difficult, notably because of spectral interference by isotopic and isomeric species [21]. Indeed, isotopic contributions from a neighboring molecular-weight fraction may be significant when there are marked differences in concentration between the two species, which is the case for ceramides and sphingomyelins [21]. Sphingomyelins formed with So (SM) are 10 to 50 times more abundant than sphingomyelins formed with Sa (DHSM), from which they differ by only one unsaturation, and quantitation is performed on the same phosphocholine head-product ion. Because long-chain fatty acids incorporated in the sphingomyelins can also be unsaturated, good chromatographic separation of the analytes is necessary during LC-MS/MS analysis for accurate quantitation of low-abundant species in MRM mode [21]. The same is true for So-derived ceramide (CER) and Sa-derived ceramides (DHCER), even if there is less interference because quantitation is performed on the product ion derived from each SB. Another limitation in the simultaneous characterization of different sphingolipids is related to the marked differences in solubility between the different classes of sphingolipids [20,22]. Marked differences in solubility are also observed within the same class of sphingolipids because of differences in the chain length of the incorporated fatty acid, which can vary from less than 10 carbons to more than 26 [20]. Consequently, a cocktail of internal standards (IS) representative of the different classes of sphingolipids is generally used for quantitation, but the precise recovery of the different analytes within the same class of sphingolipids in the different matrices to be analyzed is not always known.

The objective of this study was to investigate the effects of FB on different classes of sphingolipids in chickens fed a low dose of toxin, considered to be safe in this species [1]. In particular, the exact quantitation of the low-abundant forms of DHCER and DHSM was expected to reveal possible differences in effects related to the mechanism of action of FB. For the investigation, a highly sensitive method of quantification of 82 sphingolipids based on the optimized separation of the analytes by UHPLC-MS/MS was first developed. Next, the consequences of feeding FB for the sphingolipid profile were investigated in the liver of chickens in which an alteration in the Sa and So contents measured by HPLC with fluorescence detection had already been observed [23]. The method was also used in plasma to see whether alterations in the sphingolipid profile could be observed in the absence of an effect of FB on the Sa:So ratio, which is commonly used to characterize exposure to these mycotoxins.

## 2. Results and Discussion

### 2.1. Analysis of Sphingolipids

#### 2.1.1. Separation of the Analytes

**Figure 1:**
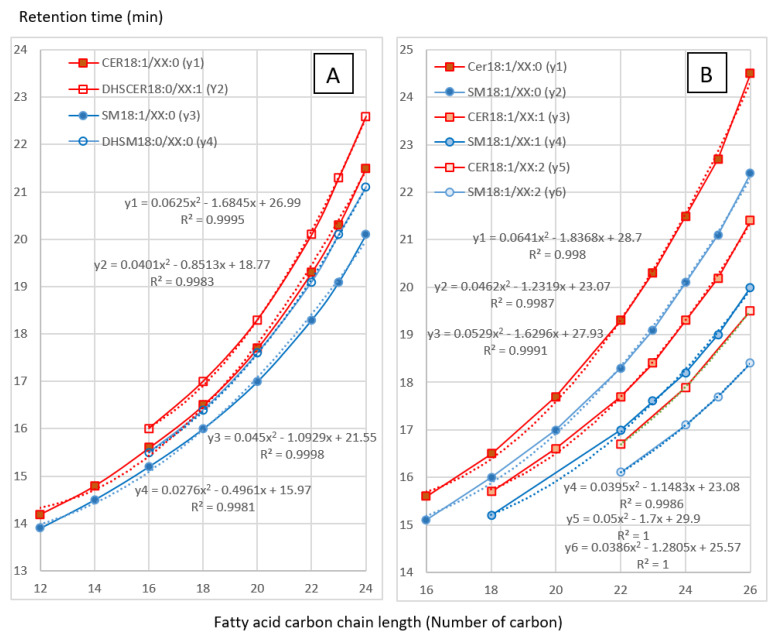
Variation of the retention time of ceramides (CER), dihydroceramides (DHCER), sphingomyelins (SM) and dihydrosphingomyelins (DHSM) in this study. A: Influence of carbon chain length on the retention time. B: Influence of unsaturation degree on the retention time. XX correspond to the number of carbon; 0, 1, and 2 correspond to the degree of unsaturation.

Retention times of the sphingolipids dosed in this study are listed in Table 1. The chromatographic conditions were selected to permit good separation of sphingolipids in order to compare the effect of FB on ceramides (CER), dihydroceramides (DHCER), sphingomyelins (SM) and dihydrosphingomyelins (DHSM). As shown in Figure 1, a very good correlation (R^2^ > 0.99) between carbon number and retention time was observed for CER, DHCER, SM and DHSM. A very good correlation (R^2^ > 0.99) was also observed between the number of unsaturations and the retention time of the analytes in the same class of sphingolipids (Figure 1). These correlations enable prediction and confirmation of the retention time of analytes that are not available as standards. All these results are in agreement with those previously described [21]. The ability to predict the retention times of sphingolipids is of particular importance for DHCER, for which only two standards are available, and for DHSM, which are not available as standards. The same goes for unsaturated and polyunsaturated CER and SM, which are not available as standards either. 

Accurate separation is important to enable fine quantitation of sphingolipids that are only present at low concentrations in biological samples. Indeed, the difference in abundance between sphingosine derivatives (d18:1), such as CER and SM, and sphinganine derivatives (d18:0), such as DHCER and DHSM, is so strong that a small [M + 2] isotopic overlap can make it difficult to identify the less abundant species in quantitative MRM analysis [21]. This problem is particularly important for sphingomyelins because SM and DHSM are identified and quantified by dosing for the same phosphocholine moiety at m/z 184. Optimization of the fragmentor energy and collision energy does not allow for differentiation between SM and DHSM. Thus, overlapping signals from isotopic [M + 2] mass ions derived from SMs can interfere with the detection of DHSM, and such interference can only be avoided by precise chromatographic separation [21]. Although sphingosine-derived ions (m/z 282.3) can be used to confirm the presence of SM, these ions are not sufficiently abundant to allow satisfactory quantitation, the ratio of m/z 184 and m/z 282.3 ions being close to 1%. The problem due to isotopic overlap is less important for ceramides because CER and DHCER can be identified by the presence of different m/z ions, respectively 282.3 and 284.3. Nevertheless, good separation between CER and DHCER was also observed with the proposed method, which enabled the accurate determination of DHCER forms [21].

#### 2.1.2. Validation of the Method on IS

Signal suppression and enhancement (SSE) measured on IS varied with the analyte and the matrix assayed (Table 2). Strong signal enhancement was observed in the two matrices for d17:0P, d17:1P and for d18:1/12:0P in liver. For other sphingolipids used, such as IS, the SSE measured ranged from 89% to 105%, indicating that no matrix interaction occurred for these analytes. Apparent recovery (RA) and recovery (R) measured for the IS in liver and plasma are presented in Table 2. Good recovery between, 70% and 120%, and low RSD, below or close to 20%, were observed for most of the 10 IS used in this study in liver and plasma. Recovery was generally higher in liver than in plasma but lower than the recovery rates previously reported in cell suspensions [21,22]. R below 70% was only observed for 18:1/25:0 in plasma, probably because of a low extraction efficiency of this analyte in this matrix. Together, the low recovery of 18:1/25:0 and interferences with the detection of 18:1/24:0 probably explain why 18:1/25:0 is not frequently used as IS [20,21,22]. R slightly above 120% was observed for d17:1P, and d17:0P in liver and d17:0P in plasma, but was considered to have only minor consequences for the estimation of sphingolipids in these two matrices. By contrast, R of 166% was observed for d18:1/12:0P in plasma, suggesting that care should be taken when using this IS to quantitate ceramides-1P in this matrix. Intraday and interday repeatability of the method was demonstrated by the low RSD measured on the IS (Table S2).

#### 2.1.3. Validation of the Method on Standards

Validation of the method using the sphingolipids available as standards was conducted on liver and plasma samples spiked at different concentrations. Because sphingolipids are normally present in liver and plasma, no blank sample free of sphingolipids was available. So, the real concentrations of standards in the spiked samples were calculated by subtracting the concentrations measured in the non-spiked sample, but other methods of validation can be used to estimate the recovery rate when the analytes to be measured are normally present in the sample to be analyzed. Some studies reported the recovery measured after extraction of artificial samples made by solubilizing the analytes in a buffer solution [21,22]. The method used in the present study was preferred because it was considered to be more representative of interactions that may occur in the complex matrices to be analyzed. For each analyte, the concentration measured in the sample was corrected by the R value obtained for the corresponding IS, as detailed in Tables S1 and S2. Each class of sphingolipids has a corresponding IS, except some analytes for which no correction was found. Concerning CER and DHCER, the R value measured for 18:1/12:0 was used to calculate the R value of long chain CER, whose carbon chain length is 18 and below. In contrast, the R value measured for 18:1/25:0 was used to calculate the R value of very-long-chain CER, whose carbon chain length is 20 and above. Indeed, the use of the R-values of 18:1/12:0 would have led to a weak recovery of very-long-chain CER with higher RSD values than those observed using 18:1/25:0. Further, the artifact previously reported when 18:1/25:0 was used as IS was not observed in the present study [20]. R values for the 33 analytes available as standards are reported in Tables S1 and S2 at the different spiked concentrations, and the mean R values obtained over the range of concentrations assayed are reported in Table 3. 

**Figure 2:**
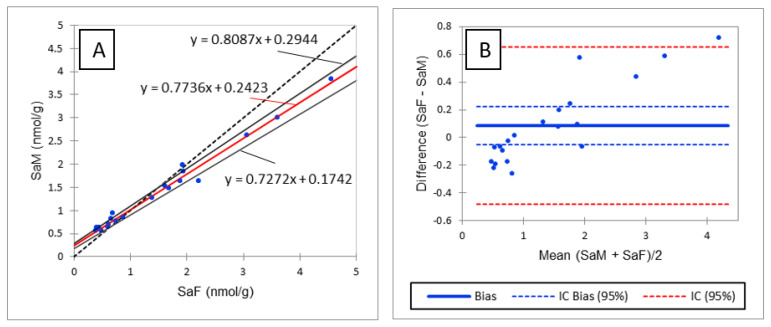
Comparison of sphinganine concentrations in liver measured by fluorescence detection (SaF) and mass detection (SaM). (**A**) Passing and Bablok regression; (**B**) Bland–Altman comparison.

As shown in Table 3, R values between 70% and 120% with RSD below or close to 20% were observed for most of the analytes assayed in liver and plasma, thus validating the method in the two matrices. R < 70% was observed for 18:1/16:0P and 18:0/24:0 in liver and SM18:1/18:1 in plasma and liver. Because the low R observed for 18:1/16:0P in liver was accompanied by an RSD of 44%, it was concluded that the method was not valid for the determination of CERP in liver. The low R values observed for SM18:1/18:1 were accompanied by an RSD of 5% and 9% in plasma and liver, respectively. It was thus concluded that even if SM18:1/18:1 was underestimated, the precision of the results remained very good. The low R value of SM18:1/18:1 in plasma and liver cannot be explained by a matrix effect, as the SSE observed for this analyte was close to the SSE observed for SM18:1/12:0 used as IS (Table 2 and Table S3). The low R value observed for 18:0/24:0 in liver is discussed below. Rs of 119% and >120% were observed for d18:0, d18:1P and d18:0P in liver and d18:1P, d18:0P and LysoSM in plasma. High R values for d18:1P and d18:0P can be explained by higher signal enhancement of these analytes in these tissues than that observed for the corresponding IS (Table 2 and Table S3). Likewise, the high R value observed for LysoSM in plasma can be explained by the signal enhancement that occurred for this analyte, for which no IS was available (Table S3). Because the low R value observed for 18:0/24:0 and the high R value observed for d18:0 in liver cannot be explained by different SSE from those observed for the corresponding IS (Table 2 and Table S3), it was hypothesized that degradation of 18:0/24:0 may occur during the extraction of sphingolipids leading to d18:0. This hypothesis was invalidated by spiking liver samples with 40,000 pmol/g of 18:0/24:0 alone, which failed to increase d18:0 content in the spiked sample. Because d18:0 is known to be the best biomarker of an effect of FB, the high recovery observed for d18:0 could be problematic when detecting an effect of FB in chickens. Consequently, the methods were compared to reveal whether similar results would be obtained with the method developed in this study and an older method in which d18:0 contents were measured by fluorescence detection after derivatization with orthophtalaldehyde [23]. As shown in Figure 2A, Passing and Bablok regression revealed a very strong linear correlation between the two methods (R² = 0.9841). However, the correlation slope appeared to deviate slightly from the bisector, suggesting a slight difference between the two methods. This hypothesis was confirmed by a Bland–Altman comparison of methods, which revealed a difference proportional to the concentration (Figure 2B). However, the bias between the two methods is so small that the means measured using the two methods did not differ (*p* > 0.05). A similar result was obtained for So (Figure S1). As the difference observed between the two methods was the same for Sa and So, the slight differences between the methods were no longer visible when the Sa:So ratio was calculated (Figure S2).

Finally, no additional correction factor other than the R value measured on the IS was used to calculate the concentrations of sphingolipids in the samples, in order to avoid introducing bias between sphingolipids available as standards and sphingolipids not available as standards. RSD values measured on standards in the recovery assays were generally less than 20% in liver and plasma. RSD values of 21%, 22% and 26% were observed for SM18:1/22:0, SM18:1/16:0, and 18:0/24:0 in liver while RSD of 21%, 22%, 24%, 27% and 27% were observed for LacSo, SM18:1/24:0, Lac18:1/24:1, Glu18:1/16:0 and Glu18:1/24:1, respectively, in plasma (Table 3). As most of these RSD were close to 20% and below 30%, they were considered as acceptable to reveal effects of FB on sphingolipids in chickens. Indeed, a strong effect of FB on sphingolipids was expected because a two-fold increase or more in SB content is generally reported at the level of 20 mg FB1+ FB2/kg feed used in this assay [23].

### 2.2. Application to Measure the Effects of FB

#### 2.2.1. Effects of FB on Sphingolipid in Liver

**Figure 3:**
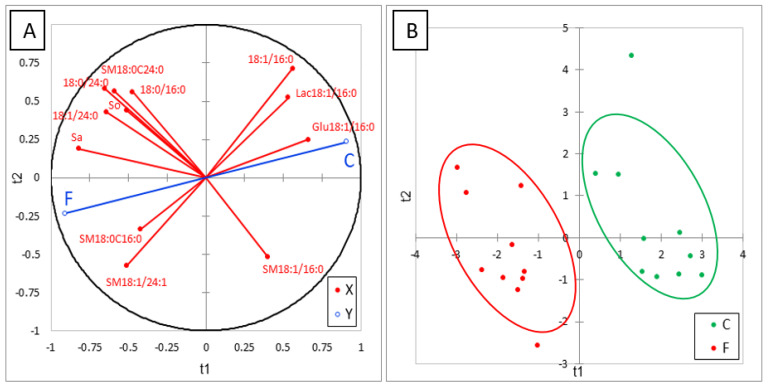
Partial least-square discriminant analysis (PLS-DA) performed on 12 sphingolipids measured in the livers of control chickens (C) not exposed to fumonisins and chickens fed for 9 days with 20 mg FB1 + FB2/kg (F). (**A**) Correlation plot between the explanatory (X) and dependent (Y) variables. (**B**) Discrimination on the factor axes extracted from the original explanatory variables. R^2^X = 0.577, R^2^Y = 0.88, Q^2^ = 0.83.

Many effects of FB on sphingolipids in liver were observed after feeding chickens 20 mg FB1 + FB2/kg for nine days (Table 4). FB increased the SB and this effect was more pronounced for Sa than So, in agreement with the literature [8]. This increase was accompanied by a marked increase in d18:1P and d18:0P. Even if d18:0P was present at very low concentrations in this study, our results are in agreement with those previously observed in ducks at higher concentrations of FB in their feed [24]. No significant difference between chickens was observed in 1dSo and 1dSa but a numerical increase in 1dSo was observed in chickens fed FB (Table 4). Additionally, a numerical increase in N-acetyl So (d18:1/2:0) was observed and N-acetyl Sa (d18:0/2:0) became detectable in chickens fed FB. Effects of FB on ceramides varied with the SB and with the length of the carbon chain. As shown in Table 4, all DHCER increased in liver of FB treated chickens, some, but not all, of these increases being significant. A significant decrease in CER occurred in d18:1/14:0 and d18:1/16:0, while a significant increase occurred in the saturated forms of CER with a chain length of 23 carbons or more. The effects of FB were less pronounced on CER whose chain length contained two or more unsaturations. By contrast, concentrations in liver of CER with a chain length of 18 to 22 carbons seem to be unaffected by feeding FB. Effects of FB on hexosyl- and lactosyl-CER also varied with the carbon chain length of the fatty acid. A significant decrease occurred in Glu18:1/16:0 and Lac18:1/16:0, while a numerically nonsignificant decrease occurred for hexosyl- and lactosyl-CER with 18-carbon chain lengths. By contrast, a significant increase in Hex18:1/24:0 occurred, while a numerical nonsignificant increase occurred in Glu18:1/24:1 and Lac18:1/24:1. Concerning sphingomyelins, an increase in SM and DHSM was observed in chickens fed FB when the carbon chain length was 18 or more. Some of these increases were statistically significant. The effects of FB were generally more pronounced on DHSM than on SM, with no apparent difference due to the carbon chain length of the fatty acid or its degree of unsaturation. A numerical nonsignificant increase also occurred in SM18:0/16:0, while a numerical nonsignificant decrease occurred in SM18:1/16:0. SM18:1/14:0 seemed to remain unaffected by FB consumption. ACP analysis of the results revealed a good correlation between most of the analytes in the same class of sphingolipids. Partial least-square discriminant analysis (PLS-DA) was conducted on 12 sphingolipids that were representative of the effect of FB (Figure 3A). This analysis clearly separated the chickens into two different groups (Figure 3B). The Q^2^ cum, R^2^Y cum, and R^2^X cum indices suggest a very good fit of the variables that are representative of the X and Y, while the confusion matrix revealed very good sensitivity and specificity of the model (Figure S3). The effects of FB on sphingolipids are discussed in the following paragraph.

#### 2.2.2. Effects of FB on Sphingolipids in Plasma

**Figure 4:**
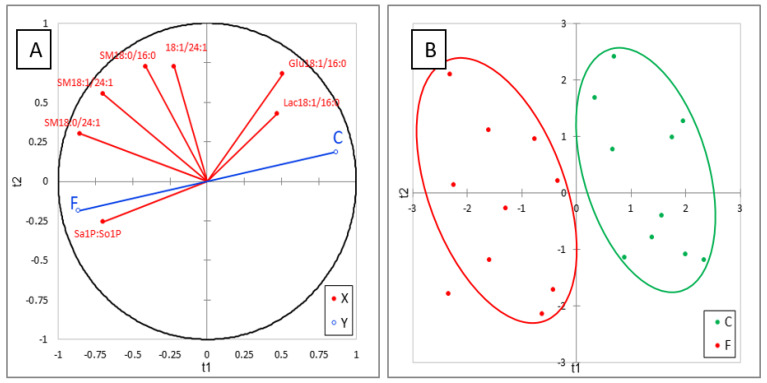
Partial least-square discriminant analysis (PLS-DA) performed on six sphingolipids and Sa1P:So1P ratio measured in the livers of control chickens (C) not exposed to fumonisins and chickens fed for 9 days with 20 mg FB1 + FB2/kg (F). (**A**) Correlation plot between the explanatory (X) and dependent (Y) variables. (**B**) Discrimination on the factor axes extracted from the original explanatory variables. R^2^X = 0.652, R^2^Y = 0.787, Q^2^ = 0.757.

The effects of FB on sphingolipids in plasma are reported in Table 4. No effect of FB on Sa and So was observed in this study, in agreement with the literature data on this level of FB in feed [23,25]. Interestingly, a significant increase in Sa1P (d18:0P) occurred, while a weak, nonsignificant increase in So1P (d18:1P) was also observed. An effect of FB on phosphorylated forms of SB has already been reported in liver of ducks for higher concentrations of FB in feed, but the present study shows for the first time that the increase in Sa1P in plasma is a more sensitive biomarker of FB than Sa [24]. No effect of FB was observed on 1dSo, 1dSa, or 18:1/2:0, which were present at very low concentrations in plasma. 18:0/2:0 was only measured in two chickens in the control group, whereas this analyte was detected in all the chickens fed the FB diet, but the concentration was very low (Table 4). No significant difference between groups was found for ceramides in plasma, DHCER were generally found at a lower concentration in chickens fed FB than in controls, while CER were generally found at a higher concentration in chickens fed FB than in controls. Additionally, no significant effect of FB on hexosyl- and lactosyl-CER was observed in plasma, but a numeric decrease was observed in Glu18:1/16:0 and Lac18:1/16:0 and a numeric increase was observed in Hex18:1/22:0, Hex18:1/24:0, and Glu18:1/24:1. Lac18:1/24:1 was not detectable in chicken plasma in this study. The effects of FB on sphingomyelins varied with the SB and with the carbon chain length. A significant decrease in SM18:1/16:0 was observed in chickens fed FB, whereas no effect was observed on SM18:0/16:0. By contrast, both SM and DHSM, whose carbon chain length of the fatty acid was 20 carbons and more, increased in chickens fed FB, and this increase was significant for a large number of analytes (Table 4). Like for liver, ACP analysis of results revealed a good correlation within the same class of sphingolipids. PLS-DA conducted on six sphingolipids, and Sa1P:So1P ratio are shown in Figure 4A. This analysis revealed the strong role of the Sa1P:So1P ratio in separating the chickens into the two groups (Figure 4B). The Q² cum, R²Y cum, and R²X cum indices and the confusion matrix reported in Figure S3 confirmed the validity of the model. PLA-DA analysis performed on the same sphingolipid plus Sa:So ratio confirmed the weak role of this ratio in plasma for discrimination of chickens fed for 9 days with 20 mg FB1 + FB2/kg feed (Figure S4).

#### 2.2.3. General Discussion of the Effects of FB on Sphingolipids

FB are responsible for dysregulation of sphingolipids in cells that varies depending on the amount of FB ingested, the organ concerned, and the duration of exposure. Because of a structural analogy of FB1 with Sa, these alterations begin by blockage of ceramide synthases and de novo synthesis of sphingolipids [13]. Six ceramide synthases have been reported in mammals [26]. Although their specificity varies with the length of the carbon chain of the fatty acid incorporated in the ceramide, all ceramide synthases are known to be inhibited by FB [8]. Inhibition of ceramide synthase leads to the accumulation of Sa in the cells and to a reduction in the production of DHCER and CER. Because ceramides are used to form glycosylsphingolipids (GSL) and sphingomyelins, inhibition of ceramide synthase also reduces the production of GSL, SM and DHSM [8]. Homeostasis of sphingolipids within the cells is complex, and the dysregulation of de novo synthesis of sphingolipids also has consequences for the recycling of sphingolipids, leading to the accumulation of So in the cells [8]. Accumulation of Sa and So increases the amounts of their phosphorylated forms Sa1P and So1P in mammals, but also in ducks [8,24]. Disruption of sphingolipid metabolism is also known to play a key role in the physiopathology of FB, although the role and importance of the different alterations in sphingolipid contents in the final expression of the disease remain largely unknown in mammals, and even less known in avian species. In this study, alterations in sphingoid bases observed in chickens fed 20 mg FB1 + FB2/kg of feed over a period of 9 days generally agreed with alterations reported in mammals. Specifically, the marked increase in Sa and the smaller increase in So, but also the accumulation of the phosphorylated forms of the sphingoid bases in liver are in agreement with results reported in mammals [8]. By contrast, only weak effects of FB were observed on 1-deoxysphingoid bases that are toxic metabolites [27]. Effects on CER, DHCER, SM, DHSM and GSL varied with the carbon chain length of the fatty acid in agreement with literature data on pigs [28]. Indeed, although C16-CER, C16-SM and C16-GSL decreased, according to the known effects of FB on ceramide synthases in mammals, C16-DHCER, and C16-DHSM increased. Moreover, not a decrease but an increase in CER, DHCER, SM, DHSM and GSL with a very-long-chain length fatty acids was generally observed. The effects of FBs on hepatic sphingolipid contents are in line with those observed in pigs, although they appear to be more pronounced in chickens [28]. Because the specificity and the expression of the ceramide synthase are not known in chickens, it is not known whether the different effects of FB observed in this study are the consequence of selective inhibition of FB on ceramide synthase or are secondary to effects on the recycling of sphingolipids. Even the decreases in C16 sphingolipids observed in this study seem to be offset by increases in C20-26 sphingolipids, this compensation is probably only apparent because ceramides have different functions depending on their chain length [29]. For example, compensatory increases in C16-ceramides and C16-sphingomyelins in cells have been reported in knockdown mice deficient in ceramide synthase 2, which is involved in the synthesis of C22 and C24-ceramides [30,31]. However, these increases only compensate for total amounts of sphingolipids in cells, and several alterations of health have been observed in knockdown mice. Finally, even if the origin of the changes in the concentrations of sphingolipids other than Sa and So observed in liver of chickens fed FB remain unknown, these changes appear to differ from those observed in mammals and could contribute the relative resistance of chickens to FB toxicity.

Concerning plasma, the effects of FB on sphingolipid contents observed in this study generally paralleled those observed in the liver. This observation is in agreement with studies in mammals [8]. This is of particular interest because the effects were observed at a nontoxic dose of FB in feed [1,23]. The sphingoid bases are membrane-permeable, but changes in Sa and So were not observed in plasma in the present study, confirming that effects of FB on the liver are easier to characterize [16,24,32]. By contrast, increased Sa1P was observed for the first time in this study in plasma of chickens fed FB. An increase in Sa1P in plasma has already been reported in mice exposed to FB1, and has been used to detect FB exposure in humans [33,34]. Because the increase in Sa1P was accompanied by alterations in GSL and SM, PLS-DA was conducted on 11 sphingolipids. This approach enabled us to distinguish all chickens exposed to FB from all unexposed controls at the individual scale. To our knowledge this study is the first to report the use of sphingolipids profiles and PLS-DA analysis for the characterization of exposure to FB. 

In conclusion, targeted analysis of sphingolipids revealed numerous alterations of sphingoid bases, ceramides, glycosylceramides, and sphingomyelins not only in liver but also in plasma of chickens fed a dose of FB considered to be safe. These alterations varied with the sphingoid base and with the carbon chain length of the fatty acid incorporated within the sphingolipid. Analysis of the phosphorylated forms of the sphingoid bases in plasma revealed exposure to FB that remained undetected by measuring Sa and So. PLS-DA of 11 sphingolipids clearly distinguished chickens fed FB from controls not exposed to the toxin.

## 3. Material and Methods

### 3.1. Analytes and Reagents

All reagents were purchased from Sharlab (Sharlab S.L., Sentmenat, Spain), and Sigma (Sigma Chemical Co, Saint Quentin Fallavier, France). Pure water, methanol, isopropanol, and formic acid were LC-MS grade; all other reagents were HPLC analytical grade. All the sphingolipids were purchased from Sigma (Sigma Chemical Co, Saint Quentin Fallavier, France). The 33 sphingolipid standards used in this study were in solid form, while the 10 sphingolipids used as internal standards were Avanti Polar Lipids “Ceramide/Sphingoid Internal Standard Mixture I” containing 25µM of sphingosine (C17 base), sphinganine (C17 base), sphingosine-1-P (C17 base), sphinganine-1-P (C17 base), lactosyl (ß) C12 ceramide, 12:0 sphingomyelin, glucosyl(ß) C12 ceramide, 12:0 ceramide, 12:0 ceramide-1-P, and 25:0 ceramide in ethanol solution.

### 3.2. Chromatographic Conditions

The 82 sphingolipids dosed in this study were analyzed according to Wang et al. (2014) with minor modifications [21]. The UPLC MS/MS system used was a binary pump with an autosampler model 1260 coupled to a triple quadrupole detector model 6410 (Agilent, Santa Clara, CA, USA). The analytes were separated on a Poroshell 120 column (3.0 mm × 50 mm, 2.7 µm) using a mobile phase composed of a mixture of solvents and an elution gradient. Solvent A was composed of methanol/acetonitrile/isopropanol (4/1/1, *v*/*v*/*v*) and solvent B was water, each containing 10 mM ammonium acetate and 0.2% formic acid (*v*/*v*). The elution gradient was 0–10 min 60–100% A, 10–30 min 100% A, and 30–35 min 60% A. The mobile phase was delivered at a flow rate of 0.3 mL/min. The injection volume was 5 µL. Detection was performed after positive electrospray ionization with the following source parameters: gas temperature: 300 °C; gas flow rate: 10 L/min; nebulizer: 25 psi; capillary voltage: 4000 V.

Table 1 lists the MRM parameters and retention times of the 82 sphingolipids dosed in this study. Transitions, fragmentor voltages, and collision energies were optimized for the different analytes available as standards using Agilent MassHunter Optimizer software. For analytes for which standards are not available, the parameters used were those of the analyte in the same class of sphingolipids with the closest mass. Different time segments were used to allow good sensitivity of the method on the 82 analytes measured. The chromatograms were analyzed using Agilent MassHunter quantitative analysis software. Quadratic calibration was used to adjust the response of the detector as a function of the amount of analyte injected using 1/x^2^ weighting factor [35]. Four replicates were performed for each concentration with a 2-fold increase between each concentration. Between 80% and 120% accuracy was observed for the majority of the analytes dosed, except for the phosphorylated forms, for which an accuracy of 65–135% was considered acceptable.

### 3.3. Extraction of Samples

The liver and plasma used in this study were obtained from a previous study in chicken [23]: 1 g of liver was homogenized in 3 ml phosphate buffer (0.1 M, pH 7.4) at 4 °C with a potter. The supernatant was collected after 15 min centrifugation at 3000× *g* and stored at −80 °C until sphingolipids extraction. Sphingolipids in liver and plasma were extracted according to Shaner et al. (2009) and Mi et al. (2016) with slight modifications [20,22]. Briefly, 40 µL of samples was diluted with 120 µL of 0.9% NaCl and placed in an ultrasonic bath for 30 s, and 600 µL of methanol/chloroform (2/1, *v*/*v*) and 10 µL of commercial solution containing the 10 IS diluted 4 times in ethanol were then added. After homogenization, samples were incubated 48 °C overnight. After cooling, 75 µL of methanol containing KOH (1M) was added and the samples were incubated for 2 h at 37 °C to cleave glycerophospholipids. Then, 8 µL of 50% acetic acid was added to neutralize KOH, and the samples were then homogenized and centrifuged at 7000× *g* for 15 min. The supernatant was collected, and the residue was extracted again with 600 µL of methanol/chloroform (2/1, *v*/*v*). The supernatant was collected, added to the previous one, and evaporated to dryness. The dry residue was solubilized in 200 µL of methanol prior to injection into the chromatographic system.

### 3.4. Linearity of the Method and Signal Suppression and Enhancement on the Internal Standards

The linearity of the method was measured by analyzing the 10 internal standards (IS) at five concentrations ranging from 78 to 1250 pmol/mL as follows: the commercial solutions containing IS, each at a concentration of 25 µmol/L, were diluted in ethanol to obtain a final concentration of 6.25 µmol/L. Variable volumes of this solution were evaporated to dryness, and the dry residue was solubilized in 200 µL of methanol prior to injection into the chromatographic system. The linear correlation between the theoretical concentration and the measured concentration of each analyte is listed in Table 2. Signal suppression and enhancement (SSE) were measured for each IS spiked in plasma and liver homogenate after extraction at 62.5 pmol/sample, equivalent to 6250 pmol/g of liver or 1563 pmol/mL of plasma. SSE was estimated for each analyte using the “one point area” method with 4 repetitions, by dividing the mean area measured in the spiked sample after extraction by the mean area measured in the neat solvent (Table 2). 

### 3.5. Recovery of the Internal Standards in Liver and Plasma

The apparent recovery (RA) and the recovery (R) of the 10 analytes used as IS were measured by dosing additions of the IS to liver and plasma before extraction, as described in paragraph 3.3. RA was calculated for each IS by dividing the area measured in the sample spiked prior to extraction by the area measured in the neat solvent. Recovery (R) was calculated by correcting the RA using the SSE previously estimated in the matrix-matched IS. RA and R are expressed in % and listed in Table 2. Intraday and interday repeatability were evaluated by the relative standard deviation (RSD) of the recovery (Table 2) in 12 samples.

### 3.6. Preliminary Estimation of Sphingolipid Available as Standard and Linearity of the Method

The concentrations of sphingolipids in liver and plasma were first measured in tissue homogenates to determine the proportion at which the sphingolipids available as standards were present. This first measurement made it possible to create a working solution (WS1) containing the 33 standards in proportions that were representative of their proportions in liver and plasma. The linearity of the method of analysis of the 33 standards was measured by evaporating variable volumes of WS1 to dryness. The ranges of concentration varied with the matrix studied to account for the marked differences in the concentration in analytes observed between plasma and liver. The ranges of concentration assayed, the slope of the calibration curves and the coefficient of determination (R^2^) obtained are reported in Tables S1 and S2. Four repetitions were performed for each concentration assayed.

### 3.7. Recovery of the Standards in Liver and Plasma

Before extraction, a variable volume of WS1 containing the 33 standards is added to liver and plasma with 10 µL of diluted IS, as described in Section 3.3; some samples were spiked with the IS alone. Recovery (R) of the IS was calculated as described in Section 3.5. The concentration of each analyte, for which a standard was available, measured in the blank sample and in the spiked sample was corrected by the R measured for the corresponding IS. The final concentration of standard in the spiked sample was obtained by subtracting the concentration measured in the unspiked sample. The R of the 33 standards at the different concentrations assayed was calculated and is reported with the RSD in Table S1 for liver and in Table S2 for plasma. Mean R and mean RSD calculated over the range of concentrations assayed are reported in Table 3.

### 3.8. Application to Sphingolipids in Samples

Because standards were not available for 29 out of the 72 sphingolipids dosed, the concentrations of these analytes in the samples were calculated using the calibration curves of the closest sphingolipids for which a standard was available. For all the sphingolipids dosed, concentrations were corrected by the recovery measured for the corresponding IS. 

### 3.9. Statistical Analysis

All statistical analyses were performed using XLSTAT Biomed (Addinsoft, 33000 Bordeaux, France). Linearity was measured using a Fisher’s test and correlation between variables were investigated with a Spearman’s test and Passing and Bablok regression. Methods were compared using Bland–Altman analysis of data. Sphingolipid in tissues is reported as mean ± SD. Groups were compared using one-way ANOVA after checking the homogeneity of variance (Hartley’s test). The quality of the partial least-squares discriminant analysis (PLS-DA) models were assessed by the R^2^ and Q^2^ (cum) values, and by the % of well-classified observations in the confusion matrix.

## Figures and Tables

**Table 1:** MRM parameters and retention time of the 82 sphingolipids dosed in this study.

Name ^1^	Category ^2^	Transition ^3^	Frag ^4^	EC ^4^	RT ^5^	Name ^1^	Category ^2^	Transition ^3^	Frag ^4^	EC ^4^	RT ^5^
1dSo	SB, S	284.5; 266.4	94	8	10.4	18:1/25:0	CER, IS	664.5; 264.3	132	32	22.7
d17:1	SB, IS	286.3; 268.4	84	4	9	18:1/26:2	CER	674.6; 264.3	132	36	19.5
1dSa	SB, S	286.5; 268.3	112	12	10.8	SM18:1/14:0	SM, S	676.0; 184.0	160	28	14.5
d17:0	SB, IS	288.3; 270.4	112	8	9.6	18:1/26:1	CER	676.6; 264.3	132	36	21.4
d18:1 (So)	SB, S	300.3; 282.3	94	4	9.9	18:1/26:0	CER	678.6; 264.3	132	36	24.5
d18:0 (Sa)	SB, S	302.3; 284.3	122	8	10.4	Glu18:1/16:0	HexCer, S	700.4; 264.3	140	30	15
18:1/2:0	CER, S	342.4; 264.3	110	24	11	SM18:1/16:0	SM, S	704.0; 184.0	190	28	15.1
18:0/2:0	DHCER, S	344.4; 266.3	120	30	11.7	SM18:0/16:0	DHSM	706.0; 184.0	190	28	15.5
d17:1P	SBP, IS	366.2; 250.3	114	12	9.2	Hex18:1/18:0	HexCER	728.4; 264.3	120	30	15.8
d17:0P	SBP, IS	368.2; 270.3	122	8	9.7	SM18:1/18:1	SM, S	730.0; 184.0	205	30	15.2
d18:1P	SBP, S	380.5; 264.3	112	12	10.1	SM18:1/18:0	SM, S	732.0; 184.0	205	30	16
d18:0P	SBP, S	382.5; 284.3	122	8	10.6	SM18:0/18:0	DHSM	734.0; 184.0	205	30	16.4
GluSo	HexCER, S	462.4; 282.3	142	20	9.3	SM18:1/20:0	SM, S	760.0; 184.0	220	32	17
LysoSM	SM, S	465.0; 184.0	140	24	9	SM18:0/20:0	DHSM	762.0; 184.0	220	32	17.6
18:1/12:0	CER, IS	482.4; 264.3	104	20	14.2	SM18:1/22:2	SM	784.0; 184.0	235	34	16.1
18:1/14:0	CER, S	510.4; 264.3	114	24	14.8	Hex18:1/22:0	HexCer	784.5; 264.3	120	30	18
18:1/16:0	CER, S	538.5; 264.3	122	24	15.6	SM18:1/22:1	SM	786.0; 184.0	235	34	17
18:0/16:0	CER, S	540.4; 284.3	140	32	16	SM18:1/22:0	SM	788.0; 184.0	235	34	18.3
18:1/12:0P	CERP, IS	562.6; 264.3	130	32	13.6	SM18:0/22:0	DHSM	790.0; 184.0	235	34	19.1
18:1/18:1	CER	564.4; 264.3	132	24	15.7	SM18:1/23:1	SM	800.0; 184.0	240	34	17.6
18:1/18:0	CER, S	566.4; 264.3	132	24	16.5	SM18:1/23:0	SM	802.0; 184.0	240	34	19.1
18:0/18:0	DHCER	568.4; 284.3	132	24	17	SM18:0/23:0	DHSM	804.0; 184.0	240	34	20.1
18:1/20:0	CER, S	594.5; 264.3	130	24	17.7	Lac18:1/12:0	LacCER, IS	806.5; 264.3	140	24	13.7
18:0/20:0	DHCER	596.4; 284.3	132	24	18.3	SM18:1/24:3	SM	810.0; 184.0	250	36	16.3
18:1/22:2	CER	618.5; 264.3	122	36	16.7	Glu18:1/24:1	HexCER, S	810.5; 264.3	100	40	18
18:1/16:0P	CERP	618.6; 264.3	130	36	14.7	SM18:1/24:2	SM	812.0; 184.0	250	36	17.1
18:1/22:1	CER	620.5; 264.3	122	36	17.7	Hex18:1/24:0	HexCER	812.5; 264.3	100	40	19.7
18:1/22:0	CER, S	622.5; 264.3	122	36	19.3	SM18:1/24:1	SM, S	814.0; 184.0	250	36	18.2
LacSo	LacCER, S	624.4; 282.3	160	28	9	SM18:1/24:0	SM, S	816.0; 184.0	250	36	20.1
18:0/22:0	DHCER	624.5; 266.3	122	36	20.1	SM18:0/24:1	DHSM	816.0; 184.0	250	36	19
18:1/:23:1	CER	634.5; 264.3	122	36	18.4	SM18:0/24:0	DHSM	818.0; 184.0	250	36	21.1
18:1/23:0	CER	636.5; 264.3	122	36	20.3	SM18:1/25:2	SM	826.0; 184.0	250	36	17.7
18:0/23:0	DHCER	638.5; 266.3	122	36	21.2	SM18:1/25:1	SM	828.0; 184.0	250	36	19
Glu18:1/12:0	HexCER, IS	644.4; 264.3	120	20	13.8	SM18:1/25:0	SM	830.0; 184.0	250	36	21.1
18:1/24:2	CER	646.5; 264.3	122	36	17.9	SM18:1/26:3	SM	838.0; 184.0	250	36	17.4
18:1/18:0_P	CERP	646.6; 264.3	130	36	15.4	SM18:1/26:2	SM	840.0; 184.0	250	36	18.4
SM18:1/12:0	SM, IS	648.0; 184.0	140	24	13.9	SM18:1/26:1	SM	842.0; 184.0	250	36	20
18:1/24:1	CER, S	648.5; 264.3	122	36	19.3	SM18:1/26:0	SM	844.0; 184.0	250	36	22.4
18:1/24:0	CER, S	650.5; 264.3	132	32	21.5	Lac18:1/16:0	LacCER, S	862.5; 264.3	180	44	14.8
18:0/24:0	DHCER, S	652.5; 266.3	190	40	22.6	Lac18:1/18:0	LacCER	890.5; 264.3	160	44	15.5
18:1/25:1	CER	662.5; 264.3	132	32	20.2	Lac18:1/24:1	LacCER, S	972.5; 264.3	100	48	17.5

^1^ GluSo = glucosylsphingosine; lacSo = lactosylsphingosine; LysoSM = lysosphingomyelin; Glu = glucosyl; Hex = hexosyl; Lac = lactosyl; P = phosphate. ^2^ SB = sphingoid base; SBP = sphingoid base-1-phosphate; CER = ceramide; CERP = ceramide-1-phosphate; DHCER = dihydroceramide; HexCER = hexosylceramide; GluCER = glucosylceramide; LacCER = lactosylceramide; SM = sphingomyelin: DHSM = dihydrosphingomyelin; S = standard; IS = internal standard. ^3^ Transition M + H^+^ (precursor ion; product ion). ^4^ Frag = energy of fragmentation (V); EC = energy of collision (V). ^5^ RT = retention time (min).

**Table 2:** Validation of the internal standard (IS) method.

	Net Solvant ^1^		Liver					Plasma				
Analyte	a	(R^2^)	SSE (%) ^2^	RA (%) ^3^	R (%) ^3^	RSD (%) ^3,4^	RSD (%) ^3,5^	SSE (%) ^2^	RA (%) ^3^	R (%) ^3^	RSD (%) ^3,4^	RSD (%) ^3,5^
d17:1	1.0001	0.9998	102	105	104	6	12	98	71	72	7	9
d17:0	1.0472	0.9962	97	98	101	6	12	96	71	74	7	7
d17:1P	0.9949	0.9992	180	228	127	8	10	168	187	111	7	17
d17:0P	0.9982	0.9994	181	219	121	7	10	163	205	126	7	14
18:1/12:0	1.0002	0.9999	102	91	90	5	8	99	87	87	5	7
18:1/12:0P	0.9965	0.9998	173	142	82	8	5	98	163	166	13	18
Glu18:1C12:0	1	0.9999	96	90	95	7	13	98	89	91	7	11
SM18:1/12:0	1	0.9999	95	82	85	7	11	100	112	112	10	11
18:1/25:0	1	1	118	84	72	22	18	105	58	55	12	22
Lac18:1/12:0	1.00002	0.9999	89	75	85	8	15	96	87	91	10	15

^1^ Measured at 5 concentrations between 78 and 1250 pmol/mL (*n* = 4 per concentration). ^2^ Measured on samples spiked after extraction with 62.5 pmol/sample equivalent to 6250 pmol/g of liver or 1563 pmol/mL of plasma (*n* = 4). ^3^ Measured on 12 samples spiked before extraction with 6250 pmol/g liver or 1563 pmol/mL plasma. ^4^ Intraday repeatability measured on 12 samples spiked before extraction with 6250 pmol/g liver or 1563 pmol/mL plasma. ^5^ Interday repeatability measured on 4 consecutive days in 3 samples per day spiked before extraction with 6250 pmol/g liver or 1563 pmol/mL plasma. a = slope of the calibration curve; R² = coefficient of determination; SSE = signal suppression and enhancement; RA = apparent recovery; R = recovery; RSD = relative standard deviation.

**Table 3:** Mean recovery measured in liver and plasma of the 33 analytes used as standards.

	Liver			Plasma		
Analyte ^1^	Range of Conc ^2^	R (%) ^3^	RSD (%) ^4^	Range of Conc ^2^	R (%) ^3^	RSD (%) ^4^
1dSo	16–250	77	7	2–31	81	8
1dSa	16–250	96	8	2–31	68	9
d18:1 (So)	5000–80,000	110	11	313–5000	115	3
d18:0 (Sa)	625–10,000	157	10	156–2500	109	8
18:1/2:0	78–1250	104	1	20–313	85	3
18:0/2:0	156–2500	116	6	39–625	86	4
d18:1P	1250–20,000	119	17	625–5000	200	11
d18:0P	1250–20,000	136	16	313–5000	263	8
GluSo	156–2500	114	8	10–156	93	7
LysoSM	78–1250	111	3	10–156	149	15
18:1/14:0	313–5000	102	14	20–313	77	11
18:1/16:0	80,000–640,000	86	3	2500–40,000	107	9
18:0/16:0	10,000–80,000	119	8	313–5000	98	10
18:1/18:0	10,000–80,000	94	16	313–5000	97	8
18:1/20:0	5000–40,000	123	6	156–2500	90	14
18:1/16:0P	5000–80,000	56	44	313–5000	100	16
18:1/22:0	40,000–320,000	81	9	1250–20,000	82	9
LacSo	39–625	104	11	10–156	110	21
18:1/24:1	40,000–320,000	79	19	2500–40,000	108	16
18:1/24:0	20,000–160,000	67	4	1250–20,000	110	10
18:0/24:0	2500–40,000	53	26	156–2500	104	18
SM18:1/14:0	156–5000	77	11	156–1250	80	12
Glu18:1/16:0	2500–40,000	84	7	313–5000	86	27
SM18:1/16:0	20,000–320,000	73	22	10,000–40,000	87	15
SM18:1/18:1	313–5000	54	5	156–2500	63	9
SM18:1/18:0	20,000–320,000	103	11	5000–80,000	81	15
SM18:1/20:0	20,000–320000	90	18	625–10,000	84	11
SM18:1/22:0	40,000–320,000	82	21	2500–40,000	88	19
Glu18:1/24:1	2500–40,000	95	13	156–2500	77	27
SM18:1/24:1	40,000–640,000	94	17	5000–80,000	82	14
SM18:1/24:0	10,000–80,000	84	15	625–10,000	85	22
Lac18:1/16:0	2500–40,000	105	15	313–5000	78	16
Lac18:1/24:1	1250–20,000	115	13	156–2500	104	24

^1^ GluSo = glucosylsphingosine; lacSo = lactosylsphingosine; LysoSM = lysosphingomyelin; Glu = glucosyl; Hex = hexosyl; Lac = lactosyl; P = phosphate. ^2^ Conc = range of concentration spiked in blank samples prior to the extraction expressed in pmo/g liver or pmol/mL plasma. ^3^ R = mean recovery calculated after correction of the concentration in spiked sample by the R measured for the IS, as explained in material and methods, Tables S3 and S4. ^4^ RSD = relative standard deviation measured over the range of concentrations assayed.

**Table 4:** Sphingolipid contents in liver and plasma of control chickens and of chickens fed a diet containing 20 mg FB1 + FB2/kg for 9 days.

Name	Liver ^1^		Plasma ^2^		Name	Liver ^1^		Plasma ^2^	
	Control	FB	Control	FB		Control	FB	Control	FB
1dSo	1.15 ± 0.24	1.76 ± 0.82	0.34 ± 0.12	0.42 ± 0.25	18:1/26:0	99 ± 71	143 ± 39	8.2 ± 7.4	8.0 ± 6.6
1dSa	10.01 ± 3.29	10 ± 4.2	0.51 ± 0.17	0.46 ± 0.18	Glu18:1/16:0	8516 ± 1397	5699 ± 1836 *	598 ± 126	452 ± 103
d18:1 (So)	7635 ± 2247	10,463 ± 2869 *	198.8 ± 84.0	184.7 ± 39.4	SM18:1/16:0	74,212 ± 17,808	59,550 ± 22,640	56,060 ± 14,141	44,320 ± 9165 *
d18:0 (Sa)	815 ± 222	2288 ± 924 *	59.0 ± 6.9	60.1 ± 8.6	SM18:0/16:0	22,531 ± 5717	30,358 ± 10,496	9961 ± 2375	11,000 ± 2849
18:1/2:0	121 ± 54	173 ± 43	12.2 ± 2.4	14.8 ± 3.1	Hex18:1/18:0	2979 ± 717	2520 ± 629	111 ± 27	112 ± 23
18:0/2:0	ND	38.7 ± 21	0.72 ± 1.44	3.60 ± 2.03	SM18:1/18:1	157 ± 49	211 ± 104	3113 ± 574	2852 ± 337
d18:1P	573 ± 191	1147 ± 419 *	1534 ± 603	1811 ± 944	SM18:1/18:0	51,624 ± 11,361	53,407 ± 16,578	17,499 ± 3123	18,240 ± 3873
d18:0P	55 ± 30	138 ± 73 *	122 ± 46	311 ± 225 *	SM18:0/18:0	5509 ± 1371	7406 ± 2279 *	751 ± 113	868 ± 147
GluSo	1344 ± 252	2082 ± 892 *	9.1 ± 3.2	9.8 ± 2.7	SM18:1/20:0	9693 ± 2262	12,346 ± 3365	1555 ± 293	1838 ± 331
LysoSM	160 ± 29	172 ± 31	31.5 ± 11.9	34.5 ± 10.3	SM18:0/20:0	1536 ± 483	2109 ± 581 *	122 ± 16	146 ± 15 *
18:1/14:0	1285 ± 226	945 ± 203 *	9.07 ± 2.60	7.65 ± 1.26	SM18:1/22:2	924 ± 234	1159 ± 303	882 ± 156	1003 ± 185
18:1/16:0	706,486 ± 124,469	504,758 ± 73,900 *	2812 ± 867	2644 ± 973	Hex18:1/22:0	8406 ± 2007	14,866 ± 4845 *	261 ± 63	304 ± 84
18:0/16:0	51,847 ± 25,043	66,188 ± 17,952	514 ± 186	421 ± 122	SM18:1/22:1	1886 ± 507	2388 ± 635	724 ± 156	819 ± 156
18:1/18:1	876 ± 170	1286 ± 328 *	97 ± 33	90 ± 22	SM18:1/22:0	126,082 ± 20,545	179,131 ± 45,760 *	6839 ± 1346	8473 ± 1611 *
18:1/18:0	75,382 ± 15,379	72,004 ± 13,639	414 ± 307	388 ± 135	SM18:0/22:0	6766 ± 3216	11,118 ± 3186 *	162 ± 33	244 ± 46 *
18:0/18:0	1796 ± 742	2610 ± 632 *	51 ± 9	57 ± 9	SM18:1/23:1	1197 ± 461	1416 ± 333	1466 ± 162	1590 ± 217
18:1/20:0	37,367 ± 11,762	44,027 ± 11,764	226 ± 137	230 ± 75	SM18:1/23:0	51,549 ± 10,638	72,012 ± 17,340 *	1814 ± 417	2482 ± 491 *
18:0/20:0	294 ± 145	446 ± 141 *	29 ± 4	30 ± 5	SM18:0/23:0	2268 ± 934	3400 ± 853 *	89 ± 13	114 ± 11 *
18:1/22:2	3724 ± 486	4220 ± 675	171 ± 20	175 ± 15	SM18:1/24:3	703 ± 148	887 ± 201 *	609 ± 92	693 ± 123
18:1/16:0P	-	-	ND	ND	Glu18:1/24:1	7094 ± 1206	10,498 ± 2830	405 ± 191	456 ± 155
18:1/22:1	9731 ± 2342	10,427 ± 1943	217 ± 47	217 ± 28	SM18:1/24:2	12,586 ± 3184	14,501 ± 4015	5324 ± 812	6157 ± 992
18:1/22:0	137,824 ± 23,336	158,290 ± 25,717	2651 ± 1440	2746 ± 1234	Hex18:1/24:0	9382 ± 2539	13,386 ± 4989 *	293 ± 69	367 ± 68
LacSo	19.6 ± 7.3	24.4 ± 10.6	ND	ND	SM18:1/24:1	58,984 ± 15,967	79,297 ± 14,028 *	10,551 ± 1717	12,718 ± 1684 *
18:0/22:0	2169 ± 567	2857 ± 420 *	205 ± 27	198 ± 23	SM18:1/24:0	19,388 ± 8080	29,423 ± 7158 *	1221 ± 229	1712 ± 264 *
18:1/:23:1	3575 ± 902	3916 ± 165	183 ± 20	176 ± 17	SM18:0/24:1	1672 ± 672	2435 ± 479 *	144 ± 20	197 ± 32 *
18:1/23:0	57,081 ± 8932	74,409 ± 16,213 *	2516 ± 909	2705 ± 787	SM18:0/24:0	1217 ± 404	1647 ± 445 *	71 ± 10	85 ± 9 *
18:0/23:0	258 ± 165	404 ± 100 *	38 ± 19	25 ± 16	SM18:1/25:2	424 ± 67	484 ± 61	121 ± 19	142 ± 15 *
18:1/24:2	141,334 ± 26,043	161,388 ± 24,647	4596 ± 2742	5429 ± 1769	SM18:1/25:1	886 ± 178	1148 ± 163 *	17 ± 30	209 ± 27 *
18:1/18:0_P	-	-	ND	ND	SM18:1/25:0	678 ± 162	922 ± 169 *	85 ± 14	98 ± 11 *
18:1/24:1	195,825 ± 22,357	208,497 ± 28,894	6237 ± 3462	6943 ± 3549	SM18:1/26:3	ND	ND	133 ± 13	145 ± 23
18:1/24:0	51,054 ± 9607	66,790 ± 13,946 *	3095 ± 1081	3209 ± 1092	SM18:1/26:2	466 ± 61	544 ± 71 *	153 ± 14	161 ± 20
18:0/24:0	2531 ± 1321	3218 ± 556	588 ± 240	480 ± 160	SM18:1/26:1	554 ± 96	640 ± 58 *	100 ± 11	109 ± 12
18:1/25:1	1260 ± 192	1685 ± 386 *	64 ± 27	77 ± 30	SM18:1/26:0	392 ± 39	433 ± 26 *	67 ± 10	73 ± 7
18:1/26:2	289 ± 85	370 ± 115	3.1 ± 4.6	4.2 ± 6.8	Lac18:1/16:0	9215 ± 2180	6374 ± 1446 *	514 ± 138	407 ± 95
SM18:1/14:0	258 ± 91	216 ± 95	579 ± 84	495 ± 90 *	Lac18:1/18:0	1953 ± 403	1875 ± 512	87 ± 27	99 ± 37
18:1/26:1	351 ± 69	449 ± 96 *	11 ± 13	23 ± 9	Lac18:1/24:1	3830 ± 1108	4639 ± 2487	ND	ND

^1^ Results are expressed in pmol/g, mean ± SD, *n* = 10. ^2^ Results are expressed in pmol/mL, mean ± SD, *n* = 10.

## Data Availability

None of the data presented have been deposited in an official repository.

## Supplementary Materials

The following are available online at https://www.mdpi.com/article/10.3390/toxins13110770/s1, Table S1: Linearity of the method in net solvent and recovery of 33 standards measured in liver; Table S2: Linearity of the method for net solvent and recovery of 33 standards measured in plasma; Table S3 Signal suppression and enhancement (SSE) of some analytes measured in liver and plasma^1^, Figure S1: Comparison of sphingosine concentrations in liver measured by fluorescence detection (SoF) and mass detection (SoM). A: Passing and Bablok regression; B: Bland-Altman comparison; Figure S2: Comparison of the sphinganine to sphingosine ratio in liver measured by fluorescence detection (SaSoF) and mass detection (SaSoM). A: Passing and Bablok regression; B: Bland-Altman comparison; Figure S3: Confusion matrix for the training sample of the PLS regression performed on variables representative of the effect of FB on SL in liver and plasma; Figure S4 Partial least square discriminant analysis (PLS-DA) performed on 6 SL, Sa:So ratio and Sa1P:So1P ratio measured in the livers of control chickens (C) not exposed to fumonisins and chickens fed for 9 days with 20 mg FB1+FB2/kg (F). A: Correlation plot between the explanatory (X) and dependent (Y) variables. B: Discrimination on the factor axes extracted from the original explanatory variables. R²X = 0.567, R²Y = 0.787, Q² = 0.735.

## Abbreviations

FB	Fumonisin B
FB1	Fumonisin B1
FB2	Fumonisin B2
Sa	Sphinganine
So	Sphingosine
SB	Sphingoid bases
CER	Ceramide formed with So
DHCER	Dihydroceramide formed with Sa
GSL	Glycosylsphingolipid
SM	Sphingomyelin formed with So
DHSM	Dihydrosphingomyelin formed with Sa
IS	Internal Standard
RSD	Relative standard deviation
SSE	Signal suppression and enhancement
R	Recovery
PLS-DA	Partial least-square discriminant analysis
