# Supplementary material for: Strong Alterations in the Sphingolipid Profile of Chickens Fed a Dose of Fumonisins Considered Safe
Source: Toxins (Basel). 2021 Oct 30;13(11):770. doi: 10.3390/toxins13110770 (PMC8619408; doi:10.3390/toxins13110770)
Supplement: Supplementary file 1 [file toxins-13-00770-s001.zip › toxins-1432139-supplementary.pdf]

toxins

MDPI

Supplementary

Strong Alterations in the Sphingolipid Profile of Chickens Fed a Dose of Fumonisins Considered Safe

Didier Tardieu [1], Maria Matard-Mann [2], Pi Nyvall Collén [2] and Philippe Guerre [1,*]

[1] National Veterinary School of Toulouse, ENVT, Université de Toulouse, F-31076 Toulouse, France; didier.tardieu@envt.fr
[2] Olmix S.A., ZA du Haut du Bois, F-56580 Bréhan, France; mmatard@olmix.com (M.M.-M.); pnyvallcollen@olmix.com (P.N.C.)
* Correspondence: philippe.guerre@envt.fr

Table S1. Linearity of the method in net solvent and recovery of 33 standards measured in liver.

Analyte[1]	Net solvant			Liver														
	Conc[2]	a	(R^2)	Conc[3]	R[4]	RSD[5]	Conc[3]	R[4]	RSD[5]	Conc[3]	R[4]	RSD[5]	Conc[3]	R[4]	RSD[5]	Conc[3]	R[4]	RSD[5]
1dSo	0.1-12	1.0833	0.9998	16	81	9	31	75	8	63	73	8	125	71	8	250	83	15
1dSa	0.1-12	1.074	0.9999	16	99	14	31	100	33	63	105	17	125	85	12	250	91	9
d18:1 (So)	125-4000	1.0015	0.9985	5000	115	34	10000	111	8	20000	111	6	40000	120	8	80000	91	12
d18:0 (Sa)	16-1000	1.077	0.9949	625	157	15	1250	169	21	2500	172	5	5000	150	4	10000	135	4
18:1/2:0	2-125	0.9911	0.9998	78	104	26	156	105	17	313	104	14	625	103	5	1250	103	4
18:0/2:0	2-250	0.9898	0.9996	156	122	6	313	106	10	625	123	18	1250	115	8	2500	115	10
d18:1_P	63-1000	0.9982	0.9994	1250	141	57	2500	103	40	5000	100	9	10000	110	13	20000	139	6
d18:0_P	63-1000	1.0155	0.9925	1250	123	16	2500	124	11	5000	119	21	10000	146	7	20000	171	6
GluSo	1-125	1.0064	0.9998	156	110	22	313	120	10	625	107	13	1250	108	13	2500	126	3
LysoSM	1-63	1.0018	0.9998	78	107	21	156	112	12	313	115	7	625	108	7	1250	113	6
18:1/14:0	4-250	0.9186	0.9975	313	119	8	625	100	8	1250	105	8	2500	107	8	5000	81	14
18:1/16:0	500-32000	1.0013	1	80000	82	19	160000	88	16	320000	89	20	640000	85	13			
18:0/16:0	125-4000	0.8859	0.9987	10000	112	11	20000	110	9	40000	129	13	80000	123	21			

18:1/18:0	125-4000	0.9913	0.9993	10000	97	8	20000	98	25	40000	108	14	80000	72	14			
18:1/20:0	125-2000	1	0.9997	5000	115	25	10000	131	19	20000	128	19	40000	119	22			
18:1/16:0_P	125-2000	1.0004	0.9996	5000	24	16	10000	40	11	20000	54	2	40000	81	17	80000	78	12
18:1/22:0	1000-16000	1.0018	0.9994	40000	82	20	80000	91	9	160000	75	12	320000	76	17			
LacSo	1-63	1.0003	0.9999	39	85	12	78	111	16	156	108	14	313	114	9	625	100	8
18:1/24:1	1000-16000	0.9834	0.9978	40000	89	23	80000	94	17	160000	67	18	320000	65	20			
18:1/24:0	125-8000	0.9897	0.9985	20000	64	14	40000	70	9	80000	67	8	160000	66	20			
18:0/24:0	8-2000	0.9918	0.9992	2500	35	22	5000	47	21	10000	53	15	20000	55	14	40000	74	6
SM18:1/14:0	2-500	0.9098	0.9974	156	71	27	625	79	10	1250	70	7	2500	72	3	5000	91	9
Glu18:1/16:0	63-4000	0.9846	0.9983	2500	81	10	5000	92	18	10000	75	14	20000	82	5	40000	87	6
SM18:1/16:0	500-16000	0.9753	0.999	20000	94	33	40000	60	16	80000	60	10	160000	86	5	320000	65	15
SM18:1/18:1	4-500	1.0131	0.9995	313	56	20	625	55	30	1250	57	6	2500	52	10	5000	51	2
SM18:1/18:0	500-32000	0.975	0.9987	20000	120	14	40000	103	6	80000	88	3	160000	100	8	320000	105	11
SM18:1/20:0	125-4000	1	0.9998	20000	112	34	40000	80	11	80000	78	3	160000	104	14	320000	78	11
SM18:1/22:0	250-16000	1.003	1	40000	69	18	80000	72	19	160000	106	19	320000	80	15			
Glu18:1/24:1	31-1000	1.0035	0.9988	2500	115	15	5000	84	29	10000	93	17	20000	98	10	40000	86	14
SM18:1/24:1	500-32000	1.009	0.9999	40000	109	32	80000	73	5	160000	80	6	320000	104	15	640000	104	12
SM18:1/24:0	63-4000	0.9926	0.9996	10000	78	27	20000	71	19	40000	98	14	80000	89	16			
Lac18:1/16:0	31-2000	1.001	0.9997	2500	118	24	5000	123	7	10000	87	15	20000	100	5	40000	96	11
Lac18:1/24:1	16-1000	0.9915	0.9993	1250	107	24	2500	139	5	5000	102	17	10000	108	8	20000	120	14

[1] Recovery (R) was calculated by correcting the concentrations measured by the R measured for each representative IS. The IS representative of the different standards were d17:1 for d18:1_dOH and d18:1, d17:1 for d18:0_dOH and d18:0; d17:1_P for d18:1_P; d17:0_P for d18:0_P; 18:1/12:0 for 18:1/14:0, 18:1/16:0, 18:0/16:0, and 18:1/18:0; 18:1/25:0 for 18:1/20:0, 18:1/22:0, 18:1/24:1, 18:1/24:0 and 18:0/24:0; SM18:1/12:0 for SM18:1/14:0, SM18:1/16:0, SM18:1/18:1, SM18:1/18:0, SM18:1/20:0, SM18:1/22:0, SM18:1/24:1, and SM18:1/24:0; Glu18:1/12:0 for Glu18:1/16:0 and Glu18:1/24:1; Lac18:1/12:0 for Lac18:1/16:0 and Lac18:1/24:1. No correction by IS was used for 18:1/2:0, 18:0/2:0, GluSo, LysoSM, and LacSo. [2] Conc = concentration expressed in pmol/mL, the ratio of each concentration tested was 2, n = 4 per concentration [3] Conc = concentration expressed in pmol/g, n = 4 [4] R = Recovery, expressed in %, n = 4. [5] RSD = relative standard deviation, expressed in %, n = 4.

Table S2. Linearity of the method for net solvent and recovery of 33 standards measured in plasma.

Analyte[1]	Net solvant			Plasma														
	Conc[2]	a	(R^2)	Conc[3]	R[4]	RSD[5]	Conc[3]	R[4]	RSD[5]	Conc[3]	R[4]	RSD[5]	Conc[3]	R[4]	RSD[5]	Conc[3]	R[4]	RSD[5]
1dSo	0.1-12	1.0833	0.9998	2	88	12	4	86	5	8	76	6	16	73	1	31	82	5
1dSa	0.1-12	1.074	0.9999	2	67	20	4	64	16	8	63	10	16	68	5	31	78	9
d18:1 (So)	16-500	0.8317	0.9905	313	111	5	625	117	4	1250	119	4	2500	114	2			
d18:0 (Sa)	8-500	0.9953	0.9998	156	115	5	313	121	4	625	107	10	1250	98	2	2500	106	12
18:1/2:0	2-125	0.9911	0.9998	20	88	10	39	85	10	78	83	4	156	84	5	313	82	6
18:0/2:0	2-250	0.9898	0.9996	39	90	11	78	90	6	156	86	8	313	81	4	625	84	5
d18:1_P	63-1000	0.9982	0.9994	625	204	27	1250	227	8	2500	194	6	5000	172	7			
d18:0_P	63-1000	1.0155	0.9925	313	260	16	625	289	7	1250	278	3	2500	231	8	5000	258	7
GluSo	1-125	1.0064	0.9998	10	96	19	20	100	10	39	86	8	78	86	6	156	97	10
LysoSM	1-63	1.0018	0.9998	10	182	10	20	162	12	39	136	9	78	134	7	156	131	8
18:1/14:0	1-63	1.0138	0.9996	20	69	13	39	72	17	78	76	10	156	75	5	313	91	12
18:1/16:0	125-8000	0.9996	0.9999	2500	119	15	5000	112	12	10000	109	6	20000	98	13	40000	96	16
18:0/16:0	16-1000	0.9954	0.9993	313	91	17	625	84	5	1250	107	13	2500	97	9	5000	108	21
18:1/18:0	16-1000	0.9945	0.9999	313	108	11	625	91	9	1250	99	12	2500	89	8	5000	99	17
18:1/20:0	8-500	0.9891	0.9997	156	78	25	313	77	18	625	98	19	1250	88	7	2500	106	19
18:1/16:0_P	125-2000	1.0004	0.9996	313	74	37	625	98	15	1250	105	16	2500	111	4	5000	113	6
18:1/22:0	63-8000	0.9864	0.9986	1250	93	11	2500	81	15	5000	78	13	10000	73	8	20000	85	22
LacSo	1-63	1.0003	0.9999	10	145	8	20	122	18	39	103	18	78	91	7	156	91	17
18:1/24:1	125-8000	0.8936	0.9961	2500	99	27	5000	121	15	10000	87	14	20000	103	19	40000	130	9
18:1/24:0	63-4000	1.0093	0.9995	1250	115	20	2500	125	8	5000	100	5	10000	98	23	20000	111	4
18:0/24:0	8-2000	0.9918	0.9992	156	129	25	313	118	12	625	101	2	1250	84	14	2500	90	13
SM18:1/14:0	2-500	0.9098	0.9974	156	88	29	313	70	15	625	73	14	1250	87	4			
Glu18:1/16:0	31-1000	0.9986	0.9999	313	124	11	625	93	14	1250	74	10	2500	63	13	5000	77	20
SM18:1/16:0	500-16000	0.9753	0.999	10000	99	23	20000	73	30	40000	88	7						
SM18:1/18:1	4-500	1.0131	0.9995	156	62	43	313	69	15	625	55	5	1250	63	11	2500	68	5
SM18:1/18:0	500-32000	0.975	0.9987	5000	97	44	10000	84	18	20000	68	17	40000	70	15	80000	85	5
SM18:1/20:0	125-4000	1	0.9998	625	93	32	1250	90	8	2500	73	12	5000	76	20	10000	89	5
SM18:1/22:0	250-16000	1.003	1	2500	113	15	5000	93	12	10000	71	13	20000	73	21	40000	90	10
Glu18:1/24:1	31-1000	1.0035	0.9988	156	103	23	313	92	21	625	72	10	1250	52	13	2500	66	11
SM18:1/24:1	500-32000	1.009	0.9999	5000	100	17	10000	78	7	20000	69	14	40000	78	18	80000	85	9
SM18:1/24:0	63-4000	0.9926	0.9996	625	112	27	1250	93	6	2500	68	21	5000	69	28	10000	82	10

Lac18:1/16:0	31-2000	1.001	0.9997	313	97	9	625	79	22	1250	80	7	2500	65	8	5000	68	16
Lac18:1/24:1	16-1000	0.9915	0.9993	156	134	19	313	124	2	625	94	13	1250	72	16	2500	98	25

[1] Recovery (R) was calculated by correcting the concentrations measured with the R measured for each representative IS. The IS representative of the different standards were d17:1 for d18:1_dOH and d18:1, d17:1 for d18:0_dOH and d18:0; d17:1_P for d18:1_P; d17:0_P for d18:0_P; 18:1/12:0 for 18:1/14:0, 18:1/16:0, 18:0/16:0, and 18:1/18:0; 18:1/25:0 for 18:1/20:0, 18:1/22:0, 18:1/24:1, 18:1/24:0 and 18:0/24:0; SM18:1/12:0 for SM18:1/14:0, SM18:1/16:0, SM18:1/18:1, SM18:1/18:0, SM18:1/20:0, SM18:1/22:0, SM18:1/24:1, and SM18:1/24:0; Glu18:1/12:0 for Glu18:1/16:0 and Glu18:1/24:1; Lac18:1/12:0 for Lac18:1/16:0 and Lac18:1/24:1. No correction by IS was used for 18:1/2:0, 18:0/2:0, GluSo, LysoSM, and LacSo. [2] Conc = concentration expressed in pmol/mL, the ratio between each concentration tested is 2, n = 4 per concentration [3] Conc = concentration expressed in pmol/mL, n = 4. [4] R = Recovery, expressed in %, n = 4. [5] RSD = relative standard deviation, expressed in %, n = 4.

Table S3. Signal suppression and enhancement (SSE) of some analytes measured in liver and plasma[1].

	Liver			Plasma		
Analyte	Concentration[2]	SSE (%)	RSD (%)	Concentration[2]	SSE (%)	RSD (%)
d18:0 (Sa)	2500-20000	66	12	313-5000	67	16
d18:1_P	2500-20000	326	6	1250-5000	287	12
d18:0_P	2500-20000	470	10	625-5000	442	13
LysoSM	80-625	162	7	39-156	188	11
18:0/24:0	1250-20000	113	2	313-5000	103	3
SM18:1/18:1	625-10000	95	3	313-2500	97	11

[1]SSE was measured using the "one point area" method as described in materials and methods for the IS. [2]Range of concentration for the analytes spiked after the extraction, expressed in pmol/g liver or pmol/ml plasma. The concentration ratio between each level tested is 2, n=4 per concentration.

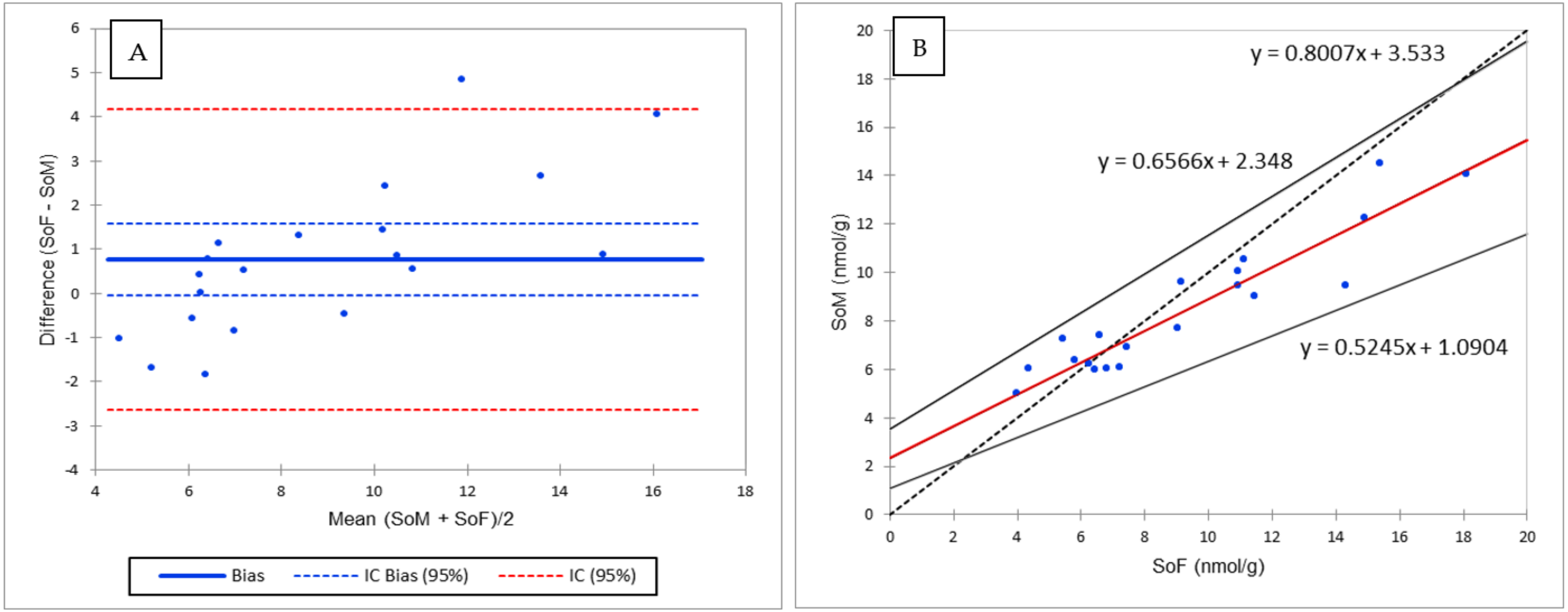

Figure S1. Comparison of sphingosine concentrations in liver measured by fluorescence detection (SoF) and mass detection (SoM). (**A**) Passing and Bablok regression; (**B**) Bland-Altman comparison.

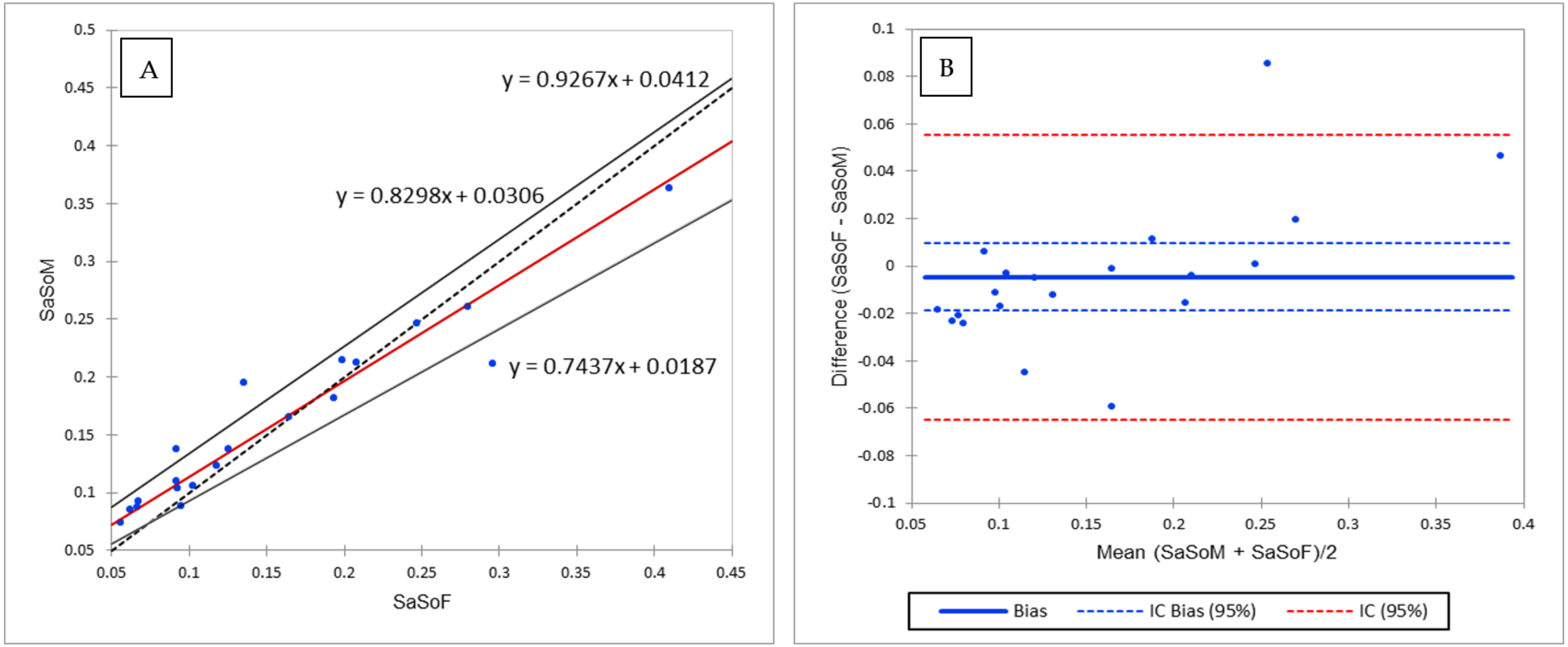

Figure S2. Comparison of the sphinganine to sphingosine ratio in liver measured by fluorescence detection (SaSoF) and mass detection (SaSoM). (**A**) Passing and Bablok regression; (**B**) Bland-Altman comparison.

Confusion matrix for the training sample (Variable Groups) : Liver				
From \ To	V	Y	Total	% correct
V	10	0	10	100.00%
Y	0	10	10	100.00%
Total	10	10	20	100.00%

Confusion matrix for the training sample (Variable Groups) : plasma				
From \ To	V	Y	Total	% correct
V	10	0	10	100.00%
Y	0	10	10	100.00%
Total	10	10	20	100.00%

Figure S3. Confusion matrix for the training sample of the PLS regression performed on variables representative of the effect of FB on SL in liver and plasma.

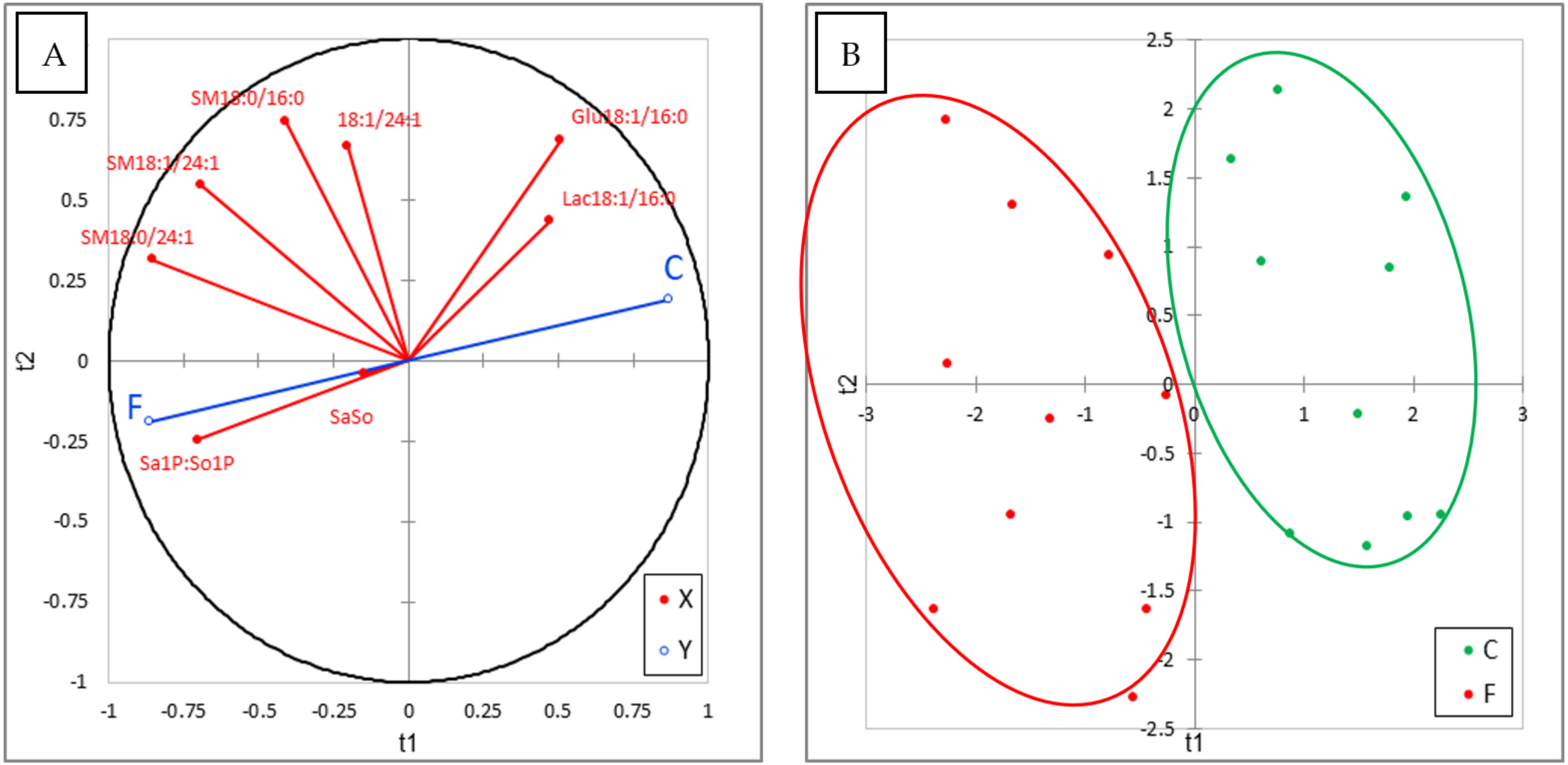

Figure S4. Partial least square discriminant analysis (PLS-DA) performed on 6 SL, Sa:So ratio and Sa1P:So1P ratio measured in the livers of control chickens (C) not exposed to fumonisins and chickens fed for 9 days with 20 mg FB1+FB2/kg (F). (**A**) Correlation plot between the explanatory (X) and dependent (Y) variables. (**B**) Discrimination on the factor axes extracted from the original explanatory variables. $R^2X = 0.567$, $R^2Y = 0.787$, $Q^2 = 0.735$.
